# Mismatch Repair Pathway, Genome Stability and Cancer

**DOI:** 10.3389/fmolb.2020.00122

**Published:** 2020-06-26

**Authors:** Nives Pećina-Šlaus, Anja Kafka, Iva Salamon, Anja Bukovac

**Affiliations:** ^1^Laboratory of Neurooncology, Croatian Institute for Brain Research, School of Medicine University of Zagreb, Zagreb, Croatia; ^2^Department of Biology, School of Medicine, University of Zagreb, Zagreb, Croatia; ^3^Department of Neuroscience and Cell Biology, Robert Wood Johnson Medical School, Rutgers University, Piscataway, NJ, United States

**Keywords:** cancer, genomic instability, microsatellite instability, MSI, mismatch repair, MMR

## Abstract

The acquisition of genomic instability is one of the key characteristics of the cancer cell, and microsatellite instability (MSI) is an important segment of this phenomenon. This review aims to describe the mismatch DNA repair (MMR) system whose deficiency is responsible for MSI and discuss the cellular roles of MMR genes. Malfunctioning of the MMR repair pathway increases the mutational burden of specific cancers and is often involved in its etiology, sometimes as an influential bystander and sometimes as the main driving force. Detecting the presence of MSI has for a long time been an important part of clinical diagnostics, but has still not achieved its full potential. The MSI blueprints of specific tumors are useful for precize grading, evaluation of cancer chance and prognosis and to help us understand how and why therapy-resistant cancers arise. Furthermore, evidence indicates that MSI is an important predictive biomarker for the application of immunotherapy.

## Introduction

While elucidated in many aspects, the molecular and genetic bases of tumor development and progression are still not completely understood. We now know that cancer is caused by distinct mutations that strike specific genes. The unique pattern of accumulated mutations in the human genome forms specific cancer molecular blueprints. Consequently, cancer today is not perceived as a single disease but a collection of diseases with specific heterogeneous genetic profiles. The genetic basis of cancer is, without a doubt, formidable because it codes for molecular changes intrinsic to a plethora of essential cellular processes. Molecular players that govern proliferation, apoptosis, differentiation, angiogenesis, cellular ability to move, as well as immune response can all be targeted in specific cancers that display unique mutational patterns (Somarelli et al., 2020). Furthermore, malfunctioning of many signaling pathways is responsible for tumor initiation and evolution. The collection of observed genetic changes is often referred to as “cancer genome landscapes” (Vogelstein et al., 2013). Our knowledge of cancer genomics is, at present, increasing almost exponentially with the ultimate goal being to understand its biology, improving diagnostic and prognostic tools and developing new therapeutic strategies that can be adjusted to individual patient needs. However, when describing essential cellular processes involved in cancer, one must not forget about the processes of DNA repair whose impaired mechanisms are also highly involved in cancer programs.

In this review we focus on microsatellite instability (MSI)—a phenomenon that belongs to genomic instability characteristic for cancer cells, and discuss it's role in cancer biology. Mismatch repair system (MMR) is responsible for maintaining genome stability. When MMR does not function normally, alterations of microsatellites occur, and the overall mutational rate of a given cell increases. Therefore, MMR has a vital role in cancer etiology and influences its biological behavior. Establishing MSI presence in cancer has significant clinical implications. It can be employed as a sensitive diagnostic tool for evaluating cancer risk, developing prognosis, and explaining how and why some cancers become resistant to chemotherapeutics. In addition, novel research shows that MSI is needed as a predictive biomarker for the application of immunotherapy.

## Mismatch Repair System (MMR) and Microsatellite Instability (MSI)

MMR is the cellular postreplication process that preserves DNA homeostasis and as such is an evolutionary guarantee of genomic stability (Kunkel, 2009). The main job of the DNA mismatch repair system is to correct spontaneous base–base mispairs and small insertions–deletion loops (indels) that are mainly generated during DNA replication. When MMR is deficient it fails to correct these errors. Consequently, the mutational rate of the cell rises as do the alterations of sequence lengths within microsatellites (Schmidt and Pearson, 2016). The variation in the lengths of the microsatellite repeats is called microsatellite instability (MSI), a type of genomic instability that is characteristic for tumor cells. Besides MSI, tumor cells can harbor another major type of instability that contributes to tumor heterogeneity called chromosomal instability, or CIN. CIN is generally associated with structural and numerical chromosomal changes (Bach et al., 2019) and is caused by errors in chromosome segregation due to abnormal alignments of chromosomes in mitotic metaphase. The high rate of chromosome segregation errors, characteristic for cancer, ultimately leads to aneuploidy in the resulting progeny of cancer cells (Sansregret and Swanton, 2017). Besides such numerical changes at the whole chromosomal level, structural chromosomal aberrations like translocations, deletions, segmental duplications, and gene amplifications are also part of CIN. Changes in common oncogenes and tumor suppressor genes are also causative of CIN, primarily by the presence of somatic mutations. Well-known examples are mutations in oncogenes myc and ras, and tumor-suppressor gene APC.

The performance of polymerases that run DNA synthesis at replication forks is not error-free. The frequency of errors committed by eukaryotic DNA polymerases is estimated at approximately one mistake for every 10^5^ nucleotides (Kunkel, 2009; Bebenek and Ziuzia-Graczyk, 2018), which means that ~100,000 errors occur during each cellular S phase. The first line of defense against such a high mutation frequency is the proofreading activity of the polymerase enzymes. Although DNA polymerases insure such lectoring activity by their own domains, some introduced mutations can still slip through unseen and need to be corrected through the second line of defense—the expression of MMR-related genes.

The mismatch repair system was originally discovered in *Escherichia coli* (Su and Modrich, 1986; Modrich, 2016). The first studies showed that mismatches in DNA molecules induce a repair reaction upon transformation into the *E. coli* cell. Later on, *E. coli* implicated genes were discovered, namely, *MutS, MutL, MutH*, and *uvrD* (ultraviolet repair protein D) (Hanaoka and Sugasawa, 2016; Modrich, 2016). It has been demonstrated that the inactivation of any of the four *E. coli* genes involved in repair increases mutation rates in the bacteria between 50- and 100-fold. Comparative studies on model organisms such as bacteria and *Saccharomyces cerevisiae* showed that elementary MMR mechanisms and proteins are highly conserved in almost all organisms ranging from bacteria to humans (Jiricny, 2013). To briefly describe the basic mechanisms of MMR in prokaryotes, it is important to understand that MMR proteins usually work as homodimers and that the cell's intact complementary DNA strand is used as a template for erroneous strand correction. The protein product of *MutS* gene initiates the MMR machinery and is responsible for mismatch detection in double stranded DNA (Sameer et al., 2014). When recognizing the lesion, the protein goes through conformational change, in order to be able to bind as a homodimer and form a stable bond with the mismatched base. After this has been accomplished, MutS recruits MutL to the MutS-mismatch complex. To be more precise, MutL acts as a mediator between the MutS and other downstream MMR effector proteins, such as MutH and UvrD (Sameer et al., 2014). Being a member of the type II family of restriction endonucleases MutH is needed for strand excision. The enzyme usually cleaves at hemimethylated GATC sites generating the so-called “nick” in the DNA which ensures the excision of a single stranded mismatch-containing DNA. DNA methylation assures that the freshly mutated DNA strand is preferentially going to be cleaved. Of note is that the mechanism of strand discrimination in eukaryotes remains uncertain. Finally, uvrD is a DNA helicase needed for unwinding DNA during recombination and works from the nick formed by MutH.

## Human Mismatch Repair Genes and Proteins

In order to mediate DNA repair, versatile proteins collectively called MSH and MLH/PMS have evolved in eukaryotes including mammals and humans. All of them fulfill their functions as heterodimers. Their names reflect the homology to the *E. coli* system, which is why name MSH is short from MutS Homolog, while MLH is derived from MutL Homolog of *E. coli*. Our knowledge of the ways in which mammalian MMR system function comes primarily from *in vitro* studies that showed that MMR mechanisms involve the following steps: lesion recognition, repair initiation, lesion excision, and DNA resynthesis (Liu et al., 2017; Huang and Li, 2018). Evidence shows that mismatch repair prefers actively transcribed genes (Huang and Li, 2018). The MMR system consists of a group of proteins that interact as heterodimers capable of perceiving and repairing mispaired bases and small loops formed from insertions or deletions. MMR repair processes have diversified in human matching to the functional combination of proteins forming the dimers. Thus, MMR machinery in humans has 8 genes that code for its components. The homologs of *E. coli MutS* genes in humans are *hMSH2, hMSH3, hMSH5*, and *hMSH6*, while *MutL* homologs are *hMLH1, hPMS1 (hMLH2), hMLH3, hPMS2 (hMLH4)* (Lipkin et al., 2000; Clark et al., 2013; Amaral-Silva et al., 2017). Variations in the deficiency of DNA repair genes are important for specific tumor susceptibility. Loss of proper functioning of DNA damage repair proteins, whether through mutations or loss of translation, introduces genomic instability critical for tumor evolution (Clark et al., 2013).

In the following paragraphs the essential functions of human MMR genes will be briefly outlined.

**Figure 1:**
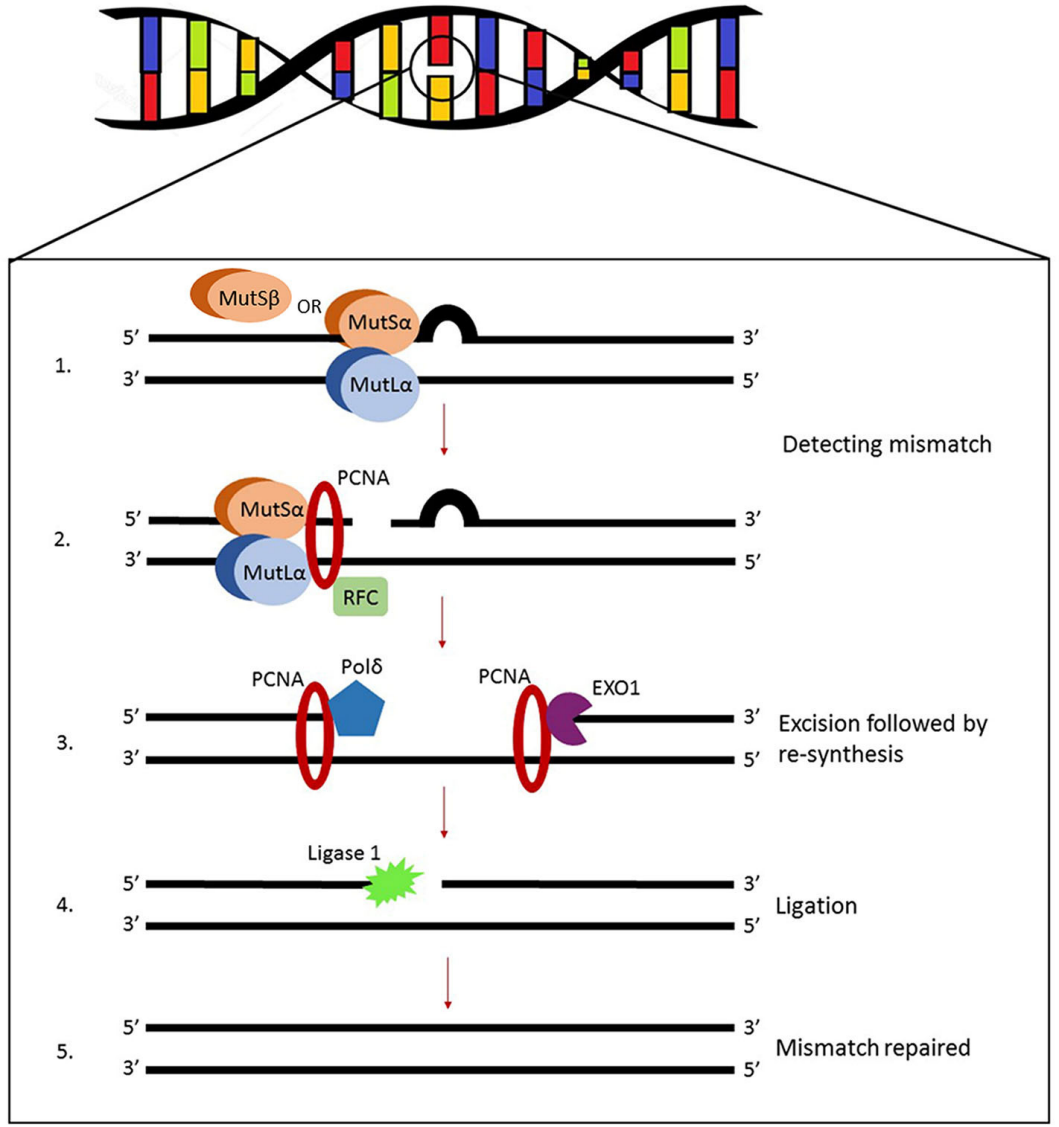
Illustration of eukaryotic MMR system. MutSα (heterodimer MSH2-MSH6 predominant in human cells) or MutSβ (heterodimer starts DNA repair by recognizing and binding to mismatches). Other molecules are recruited to the complex, primarily MutLα (heterodimer MLH1-PMS2) but also proliferating cell nuclear antigen (PCNA) and replication factor C (RFC). The assembly will initiate endonuclease activity of PMS2 which makes single-strand breaks near the mismatch and opens exonuclease 1 (EXO1) entry sites leading to the final dissociation of the DNA lesion.

The product of the gene *hMSH2*, located at chromosome 2p21, is the principal corrective MSH protein. In order to correct mispaired bases it creates two distinct heterodimers—one with MSH6, and another with MSH3. Since it has been shown that MSH6 is expressed 10 times more than MSH3 (Reyes et al., 2015), the first heterodimer predominates in human cells. Heterodimers MSH2-MSH6 and MSH2-MSH3 (also named MutSα and MutSβ, respectively) bind to mismatches while checking the postreplicated DNA strand which initiates DNA repair (Figure 1). Formed MSH complexes convert to sliding clamps on the DNA helix. They slide until mispaired bases and other extra helical lesions are recognized (Brown et al., 2016). This complex mechanism is still not fully understood and interactions of MSH proteins with the DNA, both before and after mismatch recognition, are still being interrogated by biochemical and biophysical approaches including single molecule studies (Fishel, 2015). Heterodimers MSH2-MSH6 detect single base mismatches and dinucleotide insertion-deletion distortions, while MSH2-MSH3 identify larger insertion-deletion loops that are ~13 nucleotides long (Jiricny, 2013). Their subsequent attachment to the MLH1/PMS2 complexes leads to degradation of the mutated DNA sequence fragment and the restart of synthesis.

Yet another mutS *E. coli* homolog is the *hMSH3* gene residing on chromosome 5q14.1. In a complex bound to MSH2 this protein initiates DNA repair after finding mismatches, a process which has already been described above. MMR proteins together with the DNA undergo repeating conformational changes. It has been demonstrated that following mismatch detection the MSH2-MSH3 heterodimer bends the DNA helix and that this conformational change enables the correct repair. Diseases associated with aberrations in *MSH3* gene, include colorectal, urinary bladder, and endometrial cancers (Kawakami et al., 2004; Yamamoto and Imai, 2015).

*hMSH5* is located on 6p21.3 and shows several key differences from other *MutS* homologs. It associates exclusively with MSH4 (Clark et al., 2013) and functions during meiosis in crossing-over events and gene conversions. It has been found that MSH4-MSH5 heterodimers are specifically and abundantly present in mammalian reproductive tissues since their primary role is in meiotic recombination. Although hMSH5 has not been directly implicated in MMR mechanisms, it seems that it can perform diverse cellular roles. Recent investigations have demonstrated that it is involved in the repair of double strand breaks, DNA damage response and immunoglobulin diversity, and also has important roles in both mitotic and meiotic cells. In addition, *hMSH5* SNP loci have been connected with many different human illnesses, including cancer (Clark et al., 2013).

*MutS* Homolog 6 or *hMSH6* is a member of the MMR system located on 2p16. The encoded protein is unstable until it builds a mismatch recognition complex by heterodimerization with MSH2 (Jiricny, 2013; Fishel, 2015). ATP binding and hydrolysis are vital for MMR regulation. When a G/T mismatch is recognized the MSH2-MSH6 complex exchanges ADP for ATP thus functioning as a molecular switch. A highly conserved region resides in MSH6 gene and coordinates ATP binding and hydrolysis. Sequence homology studies between amino acids revealed that MSH6 exhibits great resemblance to the human protein p160 [G/T binding protein (GTBP)] and later it has been shown that those are the same proteins (Edelbrock et al., 2013). After DNA mismatches are recognized and the first heterodimer is bound, other molecules such as proliferating cell nuclear antigen (PCNA), replication factor C (RFC), MutLα (a MLH1-PMS2 heterodimer), and exonuclease 1 (Exo1) are recruited to the complex, leading to the final dissociation of the mismatch (Liu et al., 2017). Pathogenic variants of *MSH6* are associated to Lynch syndrome type 5, cancer of the endometrium, colon and rectum (Poulogiannis et al., 2010; Rosenthal et al., 2020).

Several *MutL* homologs found in humans all belong to gyrase II/Hsp90/histidine kinase/MutL (GHKL) family of proteins that through heterodimerization are able to encircle the DNA helix (Jiricny, 2013). The first and most prevalent human *mutL* homolog, *hMLH1*, is located on chromosome 3p21. It can form heterodimers with three distinct monomers, PMS2 (postmeiotic segregation increased 2), MLH2 (also known as postmeiotic segregation increased 1, PMS1), or MLH3, which are all recruited to the MMR complex upon the first mismatch detection executed by heterodimers MSH2-MSH6 and/or MSH2-MSH3. The role of these second lines of heterodimer complexes relies primarily on the activity of MLH1-PMS2 also known as MutLα, while heterodimer MLH1-MLH3 (MutLγ) only compensates when the cell is lacking MutLα. The function of MLH1-MLH2, referred to as MutLβ, is not yet elucidated. Whether these three MMR complexes perform independently or jointly, and whether within a single or several pathways is at present still unclear. The hMLH1-hPMS2 recognizes MutSα bound to a mismatch and the assembly of this ternary complex coordinates a series of further repair steps (Kadyrov et al., 2006). The hMLH1-hPMS2 complex contains an endogenous endonuclease activity that incises the unmethylated strand. Single-strand breaks generated in this manner signal for the downstream repair processes. The nicked DNA strand near the mismatches serves as an entry point for exonuclease EXO1 which is needed for the degradation of the DNA strand containing mispaired bases (Cannavo et al., 2007; Fishel, 2015). Evidence suggests that MLH1-PMS2 interacts physically with the clamp loader subunits of DNA polymerase delta (Pol δ) and brings the enzyme to the site of the MMR. So the errors that escaped polymerase proofreading in the first place will finally be resynthesized by Pol δ (Prindle and Loeb, 2012). It has been shown that Pol α participates in the mismatch repair by interacting with MSH2-MSH6 complex (Itkonen et al., 2016). Many studies show that Lynch syndrome is caused by inactivating mutations in the *MLH1* gene. Moreover, the gene is often epigenetically silenced in a variety of cancers by CpG methylation within its promoter region, consequently not producing its polypeptide and rendering defective mismatch repair (Deng et al., 2002).

Residing on 2q31.1 human homolog, *hMLH2* is better known under the name postmeiotic segregation increased 1 (*PMS1*). As previously mentioned, the protein interacts with MLH1. In spite of the fact that intracellular quantities of PMS1 were reported to be lower than PMS2, it has been shown that PMS1 and PMS2 compete for the interaction on the same MLH1 carboxy terminal binding site (Cannavo et al., 2007). Evidence shows that mutations in PMS1 cause Lynch Syndrome type 3 either alone or merged with other MMR genes mutations (Tanakaya, 2019).

The protein product of the *MutL* homolog family member, *hMLH3*, located on 14q24.3, is present in ~40% lower molarity to PMS1 and PMS2 (Cannavo et al., 2005; Halabi et al., 2018). Still mammalian MLH3 when coupled with other family partners is primarily related to DNA insertion/deletion loops repair (Lipkin et al., 2000). Furthermore, Halabi et al. (2018) showed that one of two MLH3 isoforms—isoform 1, is responsible for GAATTC repeat expansions. MLH3 disruptions manifest in short repetitive sequence length instability. Somatic mutations of *MLH3* are often found in microsatellite instability-ridden tumors. Diseases associated with MLH3 alterations include cancer of the colon, rectum, endometrium, hereditary non-polyposis colorectal cancer type 7 (HNPCC7) and low-grade glioma (Duraturo et al., 2016; Valle et al., 2019).

*hMLH4* (*PMS2*) on 7q22.2 encodes a key MMR player. Partnering with MLH1 to form a heterodimer it plays a central role in nuclear MMR mechanisms. The hMLH1-hPMS2 interacts with MSH2-MSH6 and/or MSH2-MSH3 which initiates the endonuclease activity of PMS2, single-strand breaks will be generated and exonuclease EXO1 can perform its action (Jeon et al., 2016). Alterations in this gene are known to have a strong association with malignancies (Kasela et al., 2019). It has been shown that *PMS2* is another susceptibility gene in Lynch syndrome type 4. Although the most prevalent mutations in Lynch syndrome are confined to *MLH1* and *MSH2* genes, mutations of *PMS2* gene are also present in ~9% of the cases in this syndrome. Further mutations are found in several other conditions—colorectal cancer, Turcot syndrome, and primitive neuroectodermal tumors. It seems that MLH4 can support genome integrity at different levels, primarily through DNA repair, but also, because of its known interactions with p53 and p73, by taking a role in DNA damage-induced apoptosis (Shimodaira et al., 2003).

## Microsatellites (MS)

Repetitive DNA are immanent and innate sequence elements dispersed in our genome that account for ~3% of it. Such short repeated DNA sequences are by their nature polymorphic, and are usually known as microsatellites (MS), but can also be referred to as simple sequence repeats (SSRs). They are common across eukaryotic genomes (Ionov et al., 1993; Payseur et al., 2011; Yang et al., 2019) and their length diversity is very high ranging from mononucleotides to hexanucleotides. Microsatellite loci display great variation in population and show multiple alleles that consist of motifs usually repeated between 10 and 60 times. While the most frequently occurring repeats throughout our genome are mononucleotides, tandem repeats are also very common. They consist of dinucleotide repeats and therefore are called short tandem repeats or STRs. However, it has been reported that mononucleotides are the ones revealing the highest frequency of microsatellite instability (Payseur et al., 2011). A new phenomenon regarding genomic instability research was reported recently. It is called EMAST—short from Elevated Microsatellite Alterations at Selected Tetranucleotide repeats. This instability has been shown to occur at loci with AAAGn or ATAGn repeats (Watson et al., 2014). The knowledge on the functional role of this specific type of instability is still missing. Although the frequency of tetranucleotide repeats is generally lower in our genome than the frequency of mononucleotide repeats, reports indicate that they show increased mutation burden (Payseur et al., 2011; Watson et al., 2014). Our own work reports on constant MSI at the ATAGn locus that have been revealed in menigiomas with the marker D16S752 (Pećina-Šlaus et al., 2010).

Microsatellites are present in both prokaryotic and eukaryotic genomes. The number present in prokaryotes is low, and their amount usually correlates positively with genome size and is thus highest in mammals. It has been demonstrated that microsatellites experience higher rates of mutation when compared to other parts of the genome. The postulated causative mechanisms of high microsatellite instability are DNA polymerase slippage during replication, deficient repair processes, and unequal crossing overs (Ellegren, 2004).

The genomic distribution and allocation of microsatellites is neither evenly divided nor random (Tóth et al., 2000; Oliveira et al., 2006). Additionally, both the frequency and types of microsatellites are taxon-dependent and therefore vary in species. Data shows that, in comparison to other primates, human microsatellites contain higher numbers of repeat iterations (Oliveira et al., 2006). Microsatellites can be located in introns, coding exons, promoters, and terminal regions (Baretti and Le, 2018). However, coding and non-coding regions differ significantly in their microsatellite content, with the main difference being that non-coding regions like introns, intragenic regions and splice sites, have significantly more microsatellites. Besides coding regions, microstatellites are also less abundant in evolutionarily conserved genomic regions, like CpG islands, and transcription factor binding sites. However, microsatellites within all those regions are more stable (Sjakste et al., 2013). These findings are logical, because if coding and conserved regions where to have high MSI contents, it would reflect on the functional differences of those regions. The occurrence of MSI could seriously influence the phenotype through disturbances or loss of protein function. Meanwhile, microsatellite loci within introns or untranslated regions play roles in modulating gene expression by affecting transcription and gene splicing. The exceptions to this rule are particularly interesting microsatellites—trinucleotide microsatellites, which can be found in equal numbers in both coding and non-coding regions, with repeat motifs in multiples of three. It has been postulated that the frequency of microsatellites in coding regions is minimalized by the pressure of selection against reading-frame mutations. However, trinucleotide repeats and their expansion in the coding regions will not alter the reading frames. It is well-known that expansions of microsatellite repeats are the causative basis for some 30 developmental and neurological conditions (Sjakste et al., 2013). The prevasiveness of trinucleotide repeat expansions in neurodegenerative disorders found in man, for instance in fragile X syndrome, Huntington's disease, spinocerebellar ataxias, and myotonic dystrophies (Kim and Mirkin, 2013; Yau et al., 2018; Salcedo-Arellano et al., 2020) has been well-established. Hence, the pathogenic expansions of trinucleotide repeats are causative for many genetic diseases collectively termed repeat expansion disorders.

Another important aspect of microsatellite function is their role in chromosomal structures. Abundant repetitive DNA sequences within eukaryotic genomes are sites of heterochromatin formation. Recently it has been demonstrated that disease associated microsatellites are located in nexuses between chromatin domains (Sun et al., 2018) suggesting affected gene replication and expression. The complex molecular mechanisms that define the interplay between MMR and chromatin structure and chromatin remodeling (Goellner, 2020) are another emerging aspect of the field. Since MMR follows the footsteps of DNA polymerases in replication forks, we can assume that the two processes need to be highly coordinated. A lot of question, however, still need to be answered, for example—does MMR takes place on the naked DNA, or can it perform its actions on partially or fully reconstituted nucleosomes. Another unanswered question is whether MMR steps interact with specific histones or chromatin associated factors. Recent studies on *Xenopus* and *S. cerevisiae* describe a physical interaction between MSH2 and SMARCAD1. SMARCAD1 is the chromatin-regulatory and ATP-dependent nucleosome-remodeling protein with potential mechanistic roles in moving histones for both mispair access and excision fragment elongation. Furthermore, this protein is involved in the induction of MMR-dependent apoptosis in human cells (Takeishi et al., 2020). Another histone remodeling complex, the RSC complex, has been shown to interact with the MMR system and might also influence excision length. Chromatin assembly factor 1 (CAF1) is the histone deposition complex that plays a role in higher order chromatin condensation. CAF1 binds to PCNA and deposits newly synthesized H3/H4 histones in nucleosome making process. *In vitro* MMR studies showed that when CAF1 nucleosome assembly is incorporated into *in vitro* MMR assays, MutS alpha can repress nucleosome assembly at the mispair and slow it down sufficiently enough for efficient MMR to be performed. Additional studies showed that the MSH2-MSH6 sliding clamp can dissociate a nucleosome from DNA if a mismatch is present and that this dissociation is enhanced by H3 acetylation (Goellner, 2020). All these findings indicate the complexity of MMR and chromatin interactions and the need for further investigations.

In sum, we can conclude that microsatellites undeniably contribute to each person's DNA fingerprint. They are highly variable and informative polymorphisms that often serve as genetic markers.

## Cancer Related To MSI

In order to be able to understand MMR's role in cancer we first need to discuss the so called “mutator hypothesis.” This hypothesis has been proposed in order to rationalize the disproportion between heavily mutagenized tumor cells, and the number of mutations existing in normal cells. The usual incidence rate of spontaneous somatic mutations occuring during the lifetime of one individual does not match to the number of genetic alterations observed in tumor cells. We know now that this increased mutational extent is a consequence of genomic instability, a phenomenon that characterizes tumors. It is also well-known that carcinogenesis results from multiple sequential genetic changes. However, the mutator hypothesis relates primarily to the malfunctioning of MMR system that elicits the mutator phenotype characterized by the elevation of mutation burden. The outcome of a hypermutation phenotype is that microsatellite instability arises. Data suggest that the mutation frequency of normal human cells is much too small to explain the hundreds of genetic changes that occur above the random mutation rates. The responsibility lies in mutations of mutator genes often referred to as mutator mutations (Fishel, 2015). The mutator gene usually comes from a group of genes responsible for DNA repair mechanisms, or from the group responsible for controlling DNA synthesis fidelity. If the mutator gene is himself hit by a mutation, this will lead to increased rates of mutation in an individual's genome that cannot be properly repaired. Therefore, cancer cells displaying increased rates of genomic instability are said to comprize of a mutator phenotype. Comprehensive sequencing studies of a variety of tumors substantially supported the mutator hypothesis (Fishel, 2015). For instance, one of the largest studies supporting the mutator phenotype of human tumors is brought by The Cancer Genome Atlas (TCGA)—a project whose goal is to determine the number and type of mutations in specific tumors. TCGA reports that the number of mutations detected per tumor contrasts greatly not only to the number found in normal cells, but also between different types of tumors ranging from 500 to 100,000 mutations (Loeb, 2016).

It is generally believed that the acquisition of genetic instability is associated with progression and therefore occurs later in tumor evolution. However, there are contrasting viewpoints that suggest it may represent an early event responsible for the initiation of tumor formation. An ingenious paper (Tomasetti and Vogelstein, 2015) showed that the overall number of mitoses, stem cells carry out within a certain tissue, correlates positively with the lifetime risk of developing cancer. This suggests that the probable number of mitoses of a particular tissue should, in addition to the number of genomic instability events, be considered when evaluating the role of MSI for specific tumors. The cumulative effect of MSI and the number of cell divisions could help us better understand the difference in MSI accumulation between tumor types, as well as the specificities of cancer progression.

As they are responsible for correcting the mutational overload, MMR genes acquired the role of tumor suppressor genes. In sporadic non-heritable cancers MSI is the consequence of either inactivating mutations in one of the MMR's genes, or epigenetic mechanisms of MMR gene expression including down regulation by microRNAs (Gelsomino et al., 2016). Several papers report on MSI-associated candidate miRNAs (Jiao et al., 2017). A comparison study between miRNAs involved in colorectal cancer indicated that decreased levels of miR-552, miR-592, miR-181c, and miR-196b were observed in proficient MMR tumors as compared to increased levels of miR-625 and miR-31 in deficient MMR tumors (Sarver et al., 2009).

Studies conducted on numerous human cancers showed that hypermethylation in the promoter of the MLH1 are often associated with MSI. On the other hand, in inherited cancers, germline mutations in one or more of the MMR genes are the typical culprit for ineffective MMR and MSI. The general viewpoint on the inheritance of MSI frequency is that repeat lengths and compositions of microsatellites at specific loci are proportional to their frequencies. Although, MMR and MSI work as oncogenic drivers, it is important to understand that not every microsatellite sequence will display MSI phenotype.

**Figure 2:**
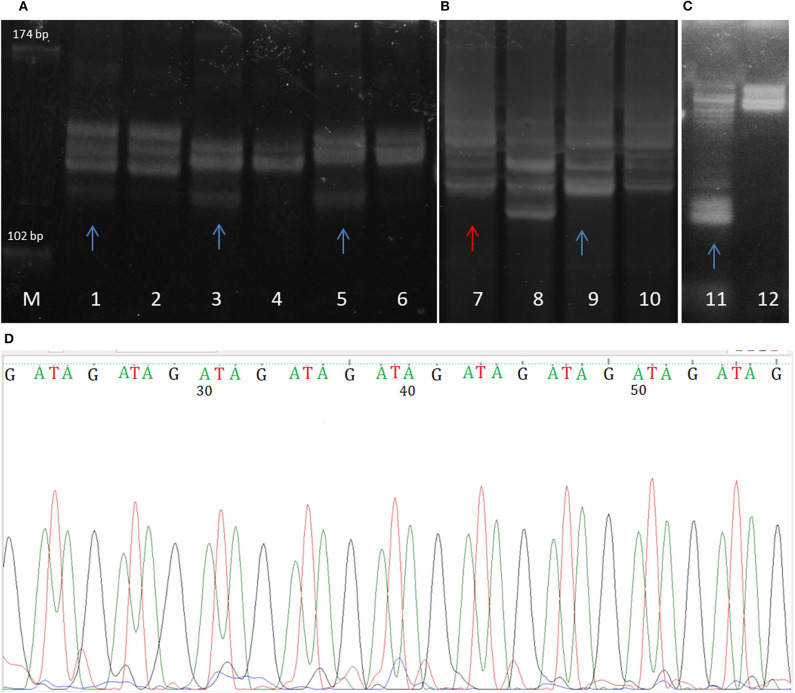
Spreadex gels showing **(A)** MSI of DVL2 gene in glioblastoma samples (microsatellilte marker D17S960); **(B)** gross deletion (lane 7) and microsatellite instability (lane 9) of MLH1 gene in mengioma samples; **(C)** MSI of the E-cadherin gene (CDH1) in meningioma. Lane M, molecular DNA standard, odd lane numbers denote tumor samples, while even numbers denote corresponding blood sample. MSIs in tumor samples are labeled with blue arrows, while sample displaying gross deletion termed loss of heterozygosity (LOH) is labeled with red arrow. **(D)** The sequence of GATA tetranucleotide repeat MSI in meningioma (microsatellite marker D16S752).

Although MSI has usually been detected by standard molecular biology methods, novel approaches in MSI diagnostics have recently been investigated and implemented. This primarily referres to the use of next generation sequencing, and the development of specific MSI sequencing panels. While these new diagnostic tools are able to detect MSI at the whole genome level, conventional diagnostic tools are still the gold standard for MSI detection in clinical testing. The classical approach for revealing lack of expression of MMR proteins is immunohistochemistry. Based on the information derived from immunohistochemistry, MSI-PCR is performed and is followed by gel or capillary electrophoresis mutation screening (Lindor et al., 2002; Modica et al., 2007). In order to reveal microsatellite instability in tumor samples, amplified DNA extracted from tumors can be pictured on PAGE or Spreadex EL gels (ALLabortechnik, Austria) after being stained with SYBR Gold (Invitrogen, Molecular Probes, USA) (Figure 2). Samples are considered MSI positive in cases when additional DNA bands appear in tumor gel lanes, or when the tumor DNA bands shift positions when compared to bands of paired blood tissue. Such inappropriate number of repeats suggests the dysfunctional DNA mismatch repair in tumor cells.

Several papers (Nowak et al., 2017; Zhu et al., 2018; Kim et al., 2019; Pang et al., 2019; Yamamoto and Imai, 2019; Rosenthal et al., 2020) report on comparisons between the classical diagnostic methods, and next generation sequencing (NSG) or targeted next generation sequencing. They show that next generation sequencing and targeted next generation sequencing can also accurately detect MSI. A recent study which compared PCR based methods with next-generation sequencing (Hempelmann et al., 2018) concluded that—the latter provided as good or even better detection. The advantages of NGS are the improvement in the speed of the analysis, and the parallel discovery of the deficient mismatch repair and its impact on the overall mutational burden of the investigated tumor. Additionally, analysis of normal control tissue is not needed for comparison. Of importance is that both approaches show high concordance levels. Recently a new tool for MSI status detection from NGS data called MANTIS was developed by Kautto et al. (2016).

As already mentioned, the longest known awareness of MSI phenotype is its involvement in colon cancer, especially Lynch syndrome or human hereditary non-polyposis colorectal cancer syndrome (Hoang et al., 1997; Umar et al., 2004; Poulogiannis et al., 2010; Sameer et al., 2014). In this common autosomal dominant syndrome occurring at a rate of 1 per 250–1,000 in the general population (Tanakaya, 2019) germline mutations in one of the mismatch repair genes are frequently identified, which eventually renders MMR system ineffective if additional mutations hit the MMR genes. The major gene responsible for Lynch syndrome is *hMLH1*. Loss of its expression happens through inactivating mutations and promoter hypermethylation, or less frequently loss of heterozygosity (Deng et al., 2002). Lynch syndrome is described with an early onset (<45 years) and tumors ridden with MSI in endometrial, gastric, renal, ovarian, or skin tissues (Hemminki et al., 1994; Pastrello et al., 2006). Studies on the expression levels of MutS homologs in a cohort of European patients suffering from sporadic colorectal carcinoma showed that all MMR genes were downregulated in those tumors (Ioana et al., 2010; Clark et al., 2013). Malfunctioning of MMR proteins, due either to mutation, or reduced expression, suggests the correlation of cancer development to the aberrations of all or the majority of MMR proteins. Besides colon tumors, many studies report on MSI in diverse malignancies including endometrial, ovarian, gastric, melanoma, prostate, lung, stomach, and glioblastoma (Leung et al., 1998; Alvino et al., 2000; Arai et al., 2016; Hause et al., 2016; Kubeček and Kopecký, 2016).

MSI serve as both predictive and prognostic marker. Current clinical guidelines recommend MSI status determination for colorectal and endometrial cancers (Bonneville et al., 2017). Large-scale analysis conducted by Bonneville et al. (2017) studied the prevalence of MSI across many additional cancer types and revealed the following numbers of MSI-H cases: 31.37% of uterine corpus endometrial carcinoma, 19.72% of colon adenocarcinoma; 19.09% of stomach adenocarcinoma, 5.73% of rectal adenocarcinoma; 4.35% of adrenocortical carcinoma, 3.51% uterine carcinosarcoma, 2.62% of cervical squamous cell carcinoma and endocervical adenocarcinoma, 2.44% pediatric high-risk Wilms tumor 2.41% of mesothelioma; 1.63% of esophageal carcinoma, 1.53% of breast carcinoma, 1.47% of kidney renal clear cell carcinoma, 1.37% of ovarian serous cystadenocarcinoma.

To standardize diagnosis for recruiting HNPCC patients the first criteria that were proposed in 1991 were called the Amsterdam Criteria by the International Collaborative Group on Hereditary Non-polyposis Colorectal Cancer. However, in 1996, at an International Workshop an improvement of these criteria including histology and genetics led to the development of the Bethesda Guidelines proposed by the National Cancer Institute (NCI) (Rodriguez-Bigas et al., 1997). These first Bethesda Guidelines recommended testing for the presence of MSI or as at that time was called RER (replication error) positive samples. A minimum of four markers were recommended to be used, with instability defined as alterations in at least two of four markers. However, the markers that were to be used could not be determined, and no consensus was reached. The panel recommended that a future workshop should be organized in order that the definition of RER, as well as the markers utilized, could be standardized. Therefore, another revision for identifying individuals with HNPCC took place—the so-called revised Bethesda Guidelines. These novel guidelines revised the original standardized microsatellite panel for CRC testing from 1998, proposed by the NCI in order to improve accuracy and sensitivity. According to the updated Bethesda guidelines (Umar et al., 2004) MSI-H applies to tumors in which MSI affects two or more of the five recommended microsatellite markers from the proposed panel that includes BAT-25, BAT-26, D2S123, D5S346, and D17S250. MSI-L characterizes tumors displaying changes in only one of the five microsatellite markers while MSS are those in which no instability is found out. The agreement on which tumor belongs to which category has been firmly defined only for colon cancers, and the classification is meaningful for predicting tumor behavior and prognosis (Zhou et al., 1997; Brennetot et al., 2005; Watson et al., 2014).

In an attempt to standardize MSI events in tumors a classification has been proposed that places tumors in category groups of MSI-High (MSI-H), MSI-Low (MSI-L), or Microsatellite-Stable (MSS). This classification depends on the amount of microsatellite markers employed that revealed MSI in tumor in comparison to patient's constitutive DNA. The principal specificity of MSI-H tumors is the increase in the number of microsatellite alleles, together with the overall numbers of unstable microsatellite loci. To simplify the classification of MSI subtypes of colorectal carcinoma, MSS and MSI-L tumors are grouped together while MSI-H is classified into another category (Mokarram et al., 2014; Watson et al., 2014; Gatalica et al., 2016). Such a brief classification is grounded on the largely similar behavior of cancers classified as MSI-L and MSS according to the total number of identified MSI events. Importantly, both groups of MSI frequencies still needs further detailed investigation.

Further research on colorectal cancer, gastric cancer, and endometrial cancer emphasizes that high MSI rate (MSI-H) tumors show a more favorable prognosis when compared to MSI-stable (MSS) or MSI low (MSI-L) ones (Yamamoto and Imai, 2015). Surprisingly the contradiction is that patients attributed with higher overall MSI burden, and diagnosed as MSI-H, show a tendency for longer survival. This could be explained with the immunotherapy response that happens in cancers with an increased mutational rate. Thus, MSI of a certain cancer is not the only responsible factor for its metastatic potential. Cancer cells that are frequent in overall MSI potential, translate larger number of mutated and truncated proteins of all kinds, which leads to the awakening of immune system that manages to slow down tumor progression (Hause et al., 2016). A novel paper indicates that mutational burden leads to an abundance of misfolded protein aggregates (McGrail et al., 2020). This explains the longer-survival-higher-MSI paradox.

An investigation by Hause et al. (2016) studied the total MSI in cancer using 5,930 cancer exomes included in the Cancer Genome Atlas (TCGA) Research Network. Eighteen different cancer types have been examined at more than 200,000 microsatellite loci. Furthermore, to determine MSI per particular unstable microsatellite loci the authors constructed a classification for genomic MSI. This comparative study revealed that cancer exomes generally number between 87 and 9,032 unstable microsatellites. Moreover, it has been shown that the average number of unstable microsatellite loci varies significantly. Colon cancers display the highest, and thyroid cancers the lowest number of MSI loci. The study has also shown that consistent MSI patterns were shared between MSI-H and MSS subjects of the same cancer type. When cancer samples were compared to normal tissue pairs it became clear that unstable microsatellites are found within, or in close proximity, to genes already renowned for their oncogenic potential. This may indicate that cancer-driving mutations are largely constituted by MSI. Thus, unstable microsatellites could be useful for recognition of new candidate cancer causing genes. In addition, MSI profiles across individual microsatellite loci demonstrated that cancers could be hierarchically clustered on the similarity in MSI signatures.

At last we would like to present our research involving MSI in human meningiomas (Pykett et al., 1994; Pećina-Šlaus et al., 2010, 2016) in which we evidenced the appearance of constant rate of MSI loci for genes *DVL3, AXIN1*, and *CDH1* in 38% of meningiomas. The presence of MSI in meningioma has been noted by other authors although in lower percentage (Sobrido et al., 2000; Chen et al., 2012; Pećina-Šlaus et al., 2017) indicating the involvement of MMR machinery. Furthermore, by using microsatellite markers D1S1611 and BAT26, we tested our cohort of meningiomas for the two major MMR genes, *MLH1* and *MSH2*, and found LOH of *MLH1* gene in 24% of investigated cases. We also established a positive correlation (*p* = 0.032) between the genetic changes of *MLH1* and *MSH2* (Pykett et al., 1994). Another study by our group investigated the presence of MSI in astrocytoma tumors using polymorphic microsatellite markers for *DVL* genes. The findings revealed that MSI was present in 28.6% of pilocytic, 61.5% of diffuse, 45.5% of anaplastic astrocytomas, and in 34.3% of glioblastomas demonstrating once again the relatively constant presence of MSI across different astrocytoma grades (Kafka et al., 2019).

Dysfunctional cellular DNA repair is very much involved in the events of cancer initiation and invasion. The findings on the many types of human tumors that exhibit MSI phenotype could be easily translated into clinic since it has been demonstrated that their MSI specificity impacts clinical behavior, recurrence, therapy effectiveness and patient survival (Clark et al., 2013; Moy et al., 2015; Thompson and Spurdle, 2015; Hause et al., 2016; Kubeček and Kopecký, 2016).

New studies also emphasize the strong link between immunotherapy and MSI phenotype (Lee et al., 2016; Willis et al., 2020). Defective MMR accompanied with MSI has become an important biomarker that can help in deciding if the specific cancer type is a good candidate for checkpoint immunotherapies. It has been shown that the application of immunotherapy is more effective in cancers with an increased mutational rate. Certain mutations that affect coding regions will result in the production of non-functional mutant proteins. Recently it has been demonstrated that as a consequence of impaired MMR and following the increased mutation burden of the tumor cell there is the parallel augmentation of the number of neoantigens. Consequently, the number of neoantigen peptides is higher in tumors with defective MMR, than in those with the correct one, and patients can benefit from immunotherapy. More precisely, the tumors that are highly immunogenic will retreat from immunotherapy (Lee et al., 2016). Neoantigenes will induce an active immune microenvironment and enhance the immunogenicity of the tumor. Endogenous cytotoxic T-lymphocytes that can recognize such neoantigens at the surface of tumor cells are going to be stimulated, thus triggering an immune response (Baretti and Le, 2018). However, immune inhibition by cytotoxic T-lymphocyte associated protein 4 (CTLA4), programmed cell death-1 (PD-1), or programmed death ligand 1 (PD-L1) that all represent immune checkpoint regulators, is also going to be recruited. For instance, it has been demonstrated that infiltrating lymphocytes are more abundant in MMR deficient colorectal cancer accompanied with the overexpression of inflammatory cytokines (Boussios et al., 2019; Liu et al., 2019). Furthermore, it has been demonstrated that multiple checkpoints, including PD-L1 and CTLA4 were up-regulated in colorectal cancer. In close connection to defective MMR another versatile biomarker is emerging—tumor mutational burden (TMB). It has also proven helpful in predicting response to immunotherapy not only to different types of immunotherapies employed but also suited for several different cancers (Duffy and Crown, 2019; Liu et al., 2019). All the evidence points to the role of MSI as an activator of innate immune signaling which might lead to progress in cancer treatments modalities that are more directed toward different responses to therapy.

Another example for MSI as a biomarker for therapeutic response is the use of chemotherapeutic drug 5-Fluorouracil (5-FU). 5-FU has been a common chemotherapy drug for advanced stages of colorectal and gastric cancer for many years (Klingbiel et al., 2015; Ilson, 2018). Guidelines from the National Comprehensive Cancer Network from 2013. recommend MSI testing for patients with stage II colorectal cancer because MSI positive patients may have a good prognosis and will not benefit from 5FU. Meta-analysis performed by Webber et al. (2015) revealed that in MSS (microsatellite stable) patients 5-FU treatment was effective and the patients had longer survival than MSS patients who were untreated. However, 5-FU treatment has not statistically significant effect on survival in microsatellite high (MSI-H) patients (Webber et al., 2015).

## Conclusions

Knowledge of the constant presence of global instability in the cancer genome teaches us that MSI plays a more important role in cancer than previously believed. Therefore, the field of genomic instability in cancer is recognized and rapidly evolving. Different frequencies of MSI are found across different malignancies, and even within a single general diagnostic category certain groups of tumors hold MSI phenotypes as a part of their genetic profile. However, it is still not clear weather dysfunctional MMR arises because of an increased need for repair that cannot keep up with the velocity of cellular DNA replication and proliferation, or whether it represents an intrinsic initiating characteristic of certain tumor cells and as such is continuously present rather than appearing later on in progression. Nevertheless, the prognostic and predictive importance of MSI for specific cancer types is nowadays regarded as an indicative biomarker of patients' response to therapy and survival. Tumors that are characterized by MMR deficiency have proven to be highly sensitive to immune checkpoint blockade. The need to quantify and standardize MSI levels in connection to the biology and behavior of the majority of tumors, still remains. It relies on future extensive prospective studies which will ultimately lead to recognition and incorporation of MSI phenotype data in hopes of improving diagnostics and treatment options in the era of personalized medicine.

## Author Contributions

NP-Š produced the idea, designed the study, wrote the manuscript, revised it for important intellectual content, and approved the final version of the manuscript. AK and AB contributed to manuscript editing, revised the manuscript for important intellectual content, and approved the final version. IS contributed to the idea, wrote the manuscript, revised it for important intellectual content, and approved the final version of the manuscript. All authors contributed to the article and approved the submitted version.

## Conflict of Interest

The authors declare that the research was conducted in the absence of any commercial or financial relationships that could be construed as a potential conflict of interest.
